# Translation and cultural adaptation of a romanian version of the communication assessment tool (CAT_Ro)

**DOI:** 10.1186/s12913-021-06186-w

**Published:** 2021-02-27

**Authors:** Andra Rodica Balanescu, Violeta Claudia Bojinca, Ana-Maria Schweitzer, Bogdan Joca, Denise Ani Mardale, Denisa Badea, Mihai Bojinca

**Affiliations:** 1 University of Medicine and Pharmacy “Carol Davila”, 37 Dionisie Lupu street, sector 2, 020021 Bucharest, Romania; 2 Department of Internal Medicine and Rheumatology, “Sf. Maria” Hospital, 37-39 Ion Mihalache Bl. Sector 1, 011172 Bucharest, Romania; 3 “Baylor Marea-Neagra” Foundation, Constanta, Romania; 4 HubCare, Bucharest, Romania; 5 Department of Internal Medicine and Rheumatology, “Dr. Ion Cantacuzino” Clinical Hospital, 5-7 Ion Movila Street, Bucharest, 030167 Romania

**Keywords:** Doctors – patients’ relationship, Communication tool, Translation, Adaptation

## Abstract

**Background:**

The communication between health providers and patients influences the quality of medical care. The Communication Skills Assessment (CAT) is a reliable, validated tool, which was developed to assess interpersonal communication skills between physicians and patients. The purpose of this study was to obtain a Romanian version of the CAT (CAT_Ro), using a controlled and systematic process to translate and cross-culturally adapt the original questionnaire, since there are no validated instruments to assess healthcare professionals’ communication capability in Romania.

**Methods:**

The study was conducted in two Departments of Internal Medicine and Rheumatology from Bucharest, Romania, using a rigorous scientific methodology for the translation process, according to literature recommendations, implicating conceptual evaluation, semantics, and cultural adaptation, which involved several steps. The updated version was pre-tested in a pilot study, which included 89 outpatients.

**Results:**

The results showed a narrow range of variability in item interpretation, without differences in patients’ responses according to variables such as age, gender, education, disease type, number of previous visits with the same doctor.

**Conclusion:**

CAT-Ro is the result of a comprehensive process study. It represents the first translation and cultural adaptation in Romanian of an instrument able to assess the health providers’ communication skills, which was validated in a pilot study and is to be used in more extensive studies with patients from several specialties.

**Supplementary Information:**

The online version contains supplementary material available at 10.1186/s12913-021-06186-w.

## Background

Modern medicine encompasses spectacular advances in understanding the pathogenic mechanisms of the diseases, the deciphering of genetic mysteries, and the development of new means of diagnosis and treatment. Beyond the scientific progress, communication between health professionals contributes substantially to the quality of medical services and increases patients’ adherence to treatment, among other benefits. In addition to professional training and scientific approach, the doctor has the social role of the healer. In this position, some communication skills play a crucial role: displaying respect, understanding, and interest for the patient and his suffering, exercising patience, compassion and interest in patients’ ideas and concerns related to own health [1, 2]. Empathy mediates effective communication around health and contributes to increasing the quality and effectiveness of the medical act [3]. The doctor-patient relationship dynamic has changed once the concept of “shared decision” between the doctor and the patient has become widespread [4].

There is a consistent body of works demonstrating that the quality of doctor-patient communication influences the observable health outcomes and the patient-reported outcomes. An analysis of the therapeutic values of doctor-patient communication indicated two paths of influence over the patient’s health. The direct path is through conversation, expression of empathy, nonverbal behavior, gestures, facial expressions, voice, which can reduce anxiety and negative emotions, increasing hope, confidence, and mental comfort of the patient. The indirect path acts through increasing confidence in the doctor and the medical system, improving adherence to treatment, self-management skills, and social support [5].

There are differences between patients’ satisfaction with the communication skills of health professionals and the medical personnel’s assessment of the same issues, the latter tending to overestimate their abilities [6]. Healthcare professionals’ attitudes are also affected when there is a divergence between them and patients or their relatives. The negative impact of conflicts can be diminished or even avoided if the patient is well informed, is involved in making choices about his health, and can explain why he disagrees with the doctor [7]. Besides, the increase in patient satisfaction is associated with a decrease in rates of malpractice complaints or claims [8].

The communication skills of physicians are recognized as vital for the quality of the medical act. Several institutions have included training and assessment of physicians’ communication skills as one of the mandatory skills and competencies for physicians. However, defining and evaluating communication skills is not easy [9]. The Can MEDS Framework defines communication skills as a combination of the following behaviors:

1. Establishing professional therapeutic relationships with patients and their families
2. Eliciting and synthesizing accurate and relevant information, incorporating the perspectives of patients and their families
3. Sharing health care information and plans with patients and their families
4. Engaging patients and their families in developing plans that reflect the patient’s health care needs and goals
5. Documenting and sharing written and electronic information about the medical encounter to optimize clinical decision-making, patient safety, confidentiality, and privacy [10].

For the above reasons, many training programs for health professionals focus on communication with patients.

The SEGUE Framework is one of the most popular, well-known, and sophisticated tools for learning and evaluating communication skills. The acronym comes from **S**tage set, **E**licit information, **G**ive information, and **U**nderstand the patient’s perspective, **E**nd the encounter [11]. It contains a list of 32 items of patient-doctor communication tasks, whose acceptance, validity, and reliability have been confirmed in numerous scientific papers or clinical practice, in medical or academic units, and various specialties. A limitation of this tool is that it is a checklist (the answer for each item is YES or NO) and is less useful in measuring the increase in communication skills compared to a ranking scale. Another example is a program for dermatologists structured upon generally valid principles, such as: organizing the agenda for the current visit, ensuring reflective listening, using the NURS approach (**N**ame, **U**nderstanding, **R**espect, a**S**sistance), using various and easy-to-understand methods to provide information to patients, checking if the patient has understood (using the teach-back technique). Other communication principles included by the above program are: using teaching tools such as written worksheets, audio-visual aids, and internet materials, and combining them with verbal instructions, making sure to resolve all issues and questions before making a shared decision with the patient [12, 13]. These programs are not homogeneous, and there is no consensus on tools for assessing physicians’ ability to communicate [14].

A systematic review of the literature on existing tools for assessing physicians’ communication skills, which looked at 45 instruments in 57 studies, found an extensive heterogeneity between the tools used, highlighting the lack of consensus [7]. This analysis concluded that it is necessary to develop unitary training methods for health professionals and to use assessment tools that are easy to manage, simple, time-sparing, standardized, validated, easy to reproduce, and generally accepted by users.

Many instruments mix the communication elements with aspects related to patients’ satisfaction with medical activity, the duration of the evaluation period is different (last consultation or longer periods), the evaluation is done in various conditions (outpatient or hospitalization). For example, there is a validated tool designed exclusively to assess how satisfied patients are with the information they receive about the prescribed medications: the “Satisfaction with Information about Medicines Scale” (SIMS) [15].

Patients’ ability to understand and communicate is influenced by many factors: their disease status, genetic predisposition, personality, education, medical knowledge, anxiety, overestimated expectations, and coping style.

A study conducted in 31 European countries, which used a patient-generated questionnaire (PCVq) and also analyzed aspects related to the cultural and socio-economic dimensions, showed that patients most appreciate being treated as partners and continuity in medical care. At the same time, most disliked was the need to obtain additional information and to prepare before a visit [16].

One of the essential factors in establishing excellent communication with patients is time. The duration of the visit can influence the establishment of a deeper and more complex relationship with the patient. Duration depends on many factors, including the complexity of the patient’s pathology, age, and psycho-intellectual condition. However, more time does not guarantee better communication, the proper use of time being essential in this regard. An analysis of published data on the efficacy of communication in the doctor-patient relationship indicated three areas of improvement for the effective use of communication: developing a friendly relationship with patients, establishing a consultation plan, and acknowledging social or emotional clues with empathy [17].

The Communication Skills Assessment (CAT) was developed to assess interpersonal communication skills between physicians and patients. This tool’s development process has been complex, and its reliability, validity, and feasibility confirmed in several studies. Since 2009, the Accreditation Council for Graduate Medical Education (ACGME) has recommended this tool for evaluating young physicians in the USA [18]. The original CAT article describes all the details related to development procedures and psychometric parameters [19]. This tool assesses patients’ opinions shortly after hospitalization or outpatient visit regardless of the clinical specialties. It is a reliable and valid questionnaire for measuring patients’ opinions in connection with the doctor’s ability to communicate [20–22]. CAT consists of 15 items measured on a 5-point response scale (1 = poor; 2 = fair; 3 = good; 4 = very good; 5 = excellent). The application alternatives are several: on paper, over the telephone, or on the internet.

Furthermore, it can be self-administered or administered by an interviewer, since its completion does not take long, and there are no restrictions related to patients’ age, sex, race, or education level. Both doctors and patients can use it, and it is useful in daily practice (on the first visit or subsequent visits), and for research purposes. Interpreting and analyzing the results is simple, and there is a section where the patients can rate global care and another one designated to patients’ comments.

Currently, there are no validated instruments to assess patient - healthcare professionals’ communication (HCP) ability in the Romanian healthcare system. This study uses a controlled and methodical process to translate and cross-culturally adapt a Romanian version of the Communication Assessment Tool (CAT_Ro).

## Methods

### Ethics and consent to participate

The study was approved by Ethics Committee of “Sfanta Maria” Clinical Hospital number 2180/06.02.2020. The patients were included in the study after they agreed and signed a written informed consent.

The process of translation and cross-cultural adaptation of the CAT in Romanian was performed according to internationally accepted and recommended guidelines of International Society of Pharmacoeconomics and Outcome Research (ISPOR), data from international literature, and recommendations made by the World Health Organization (WHO) about the process of translation and adaptation of instruments [23–25]. Moreover, a similar method, based on the same internationally accepted guidelines was used for the translation and cultural adaptation of the Italian version of the CAT by Scala and colleagues [26].

### Aim, design and setting of the study

Taking into consideration that the questionnaire is addressed to different types of patients, with various educational and cultural backgrounds, the present study aim is to ensure cross-cultural and conceptual correspondence rather than simple linguistic/literal equivalence [24].

A working group was established for the development of this tool, including five doctors, one pharmacist, one psychologist, one patient-HCP communication expert, one forward translator; all group members were bilingual Romanian/English.

### Description of all processes, interventions and comparisons

The process was carried out systematically and involved several steps:

- getting the permission to translate the CAT from his author, Professor Gregory Makoul;
- elaboration of the protocol and patients’ written informed consent;
- obtaining the hospitals’ Ethics Committee approval;
- recruitment and training of translators (e.g., to use the conceptual translation of a word or phrase, to offer a simple, clear, and concise version, to avoid the use of any jargon or terms that cannot be comprehended by ordinary people in everyday life, regardless of their age, education, and cultural background);
- development independent translations from English to Romanian (performed by four of the working group members and an independent translator, educated in the English-speaking culture, but not native English speakers);
- backward translation of the reconciled Romanian version into English (performed by an independent translator, native in English, who fluently speaks Romanian, and who did not have any connections with the questionnaire), as a quality-control step to make sure that the translation maintains the same meaning of each statement of the survey;
- harmonize the previous CAT_Ro version after comparing the original English version to the back-translated one and obtaining the second CAT_Ro version;
- develop a pilot study to test this CAT_Ro version on a group of 59 patients and obtain the third CAT_Ro version after harmonization with the patients’ comments and observations [27];
- testing the refined third CAT_Ro version on another 30 patients, following the same methodology used previously, and obtaining the final CAT_Ro.

After each phase, the working group’s members identified and resolved the translation’s inadequate terminologies, as well as any inconsistencies between the forward translation and the present or previous versions of the issue, discussed word meanings and suggested alternatives, until consensus was achieved. The study team ensured the documentation of all the discussions.

### Characteristics of participants

The first part of the pilot study included 59 patients from two Departments of Internal Medicine and Rheumatology from Bucharest, Romania. We successively included patients who were Romanian native speakers from the outpatient department, after they agreed to participate in the study and signed the informed consent. We excluded from enrollment only patients not willing to sign the informed consent and patients with cognitive deficits.

The questionnaires and the discussions with the patients were conducted by two doctors, not involved in the patients’ care. Additionally, they were trained in the interview techniques by a psychologist with expertise in the field.

We structured the cognitive interviews around the following issues: if there are any words or expressions participants did not understand, found unacceptable or offensive, what they presumed the item was asking, whether they could replicate its content in their own words, what came to their mind when they heard a specific expression or word. Participants were invited to provide alternative words or phrases, which they considered being more appropriate, or choose between several options to get a better version, more suitable to their everyday language.

All the patients’ comments and suggestions during this pilot study were discussed and analyzed item-by-item by the working group, who identified and made minor translation modifications necessary for improvement.

### Type of statistical analysis used

The statistics of choice were descriptive; we assessed frequencies and percentages to obtain the overall image of the demographics and other patient characteristics, as well as for the global item response distribution. Reliability was tested through the analysis of internal consistency using SPSS v24. The overall scale reliability was assessed by both variants of statistical Cronbach test (Cronbach’s alpha and Cronbach’s Alpha Based on Standardized Items).

The CAT_Ro refined version was then obtained and was validated on another group of 30 patients following a similar methodology. No other changes were necessary after this last phase, so the final CAT_Ro, Copyright© 2020 Gregory Makoul, PhD’ was obtained.

## Results

To evaluate if the harmonized Romanian version (CAT-Ro) of the questionnaire was easy to understand, a comprehension test was carried out, organized in two parts. The first group included 59 patients. Following another harmonization round, the refined version was further administrated to a second group consisting of 30 patients. The questionnaire was administered by two doctors who were not involved in the patients care and who acted as interview operators. The operators were previously trained in the process of interviewing by a psychologist with expertise in the field.

The demographic characteristics of the two groups (including 89 patients) were obtained and analyzed together (sex, age, diagnosis, ethnicity, education background) (Table 1).

**Table 1:** Demographic Characteristics of Patients (*n* = 89)

	n	%
**Sex**		
Women	56	37.1
Men	33	62.9
**Age**		
50.63 ± 13.78 (mean ± SD; years)		
18–24	2	2.2
25–44	29	32.6
45–64	39	43.8
65–79	19	21.3
**Urban dwelling (n, %)**	72	80.9
**Native Romanian Speaker**	89	100
**Number of previous visits with the physician**		
none	15	16.9
one	8	9
two or more	66	74.2
**Education**		
higher (above high-school level)	44	49.4
high or assimilated	40	45
basic	5	5.6
**Chronic disease**	86	96.6

In order of data analysis, the statistics of choice were descriptive through frequencies and percentages used to obtain the overall image on the demographics and other patient characteristics, as well as for the global item response distribution.

Similar to other studies, we have interpreted all “excellent” responses as a passed item and all other responses as a failed item, indicative of some room for improvement in communication [26].

The distribution of the percentage of excellent ratings for individual CAT items is presented in Table 2. This table indicates that the range of variability in item responses was quite narrow in the samples evaluated. The range observed was between 88.8 and 96.6. The items with highest scores were items 12 and item 3, and those with a slight indication for the need for improvement were items 10, item 11, 14, and item 1. Yet, since the current study focuses on translation and cultural adaptation, no general recommendations for current practice can be formulated as such.

**Table 2:** Percentage of excellent ratings for individual CAT items

	Ratings (% Excellent) ***N*** = 89
1. Greeted me in a way that made me feel comfortable	91
2. Treated me with respect	94.4
3. Showed interest in my ideas about my health	96.6
4. Understood my main health concerns	92.1
5. Paid attention to me (looked at me, listened carefully)	95.5
6. Let me talk without interruptions	93.3
7. Gave me as much information as I wanted	93.3
8. Talked in terms I could understand	94.4
9. Checked to be sure I understood everything	95.5
10. Encouraged me to ask questions	88.8
11. Involved me in decisions as much as I wanted	91
12. Discussed next steps, including any follow-up plans	98.9
13. Showed care and concern	93.3
14. Spent the right amount of time with me	91
15. How the team treated the patient	93.3

Reliability was tested through the analysis of internal consistency using SPSS v24. Results indicate that the overall scale reliability is high for the 14-items of the Romanian CAT version for both variants of statistical Cronbach test (Cronbach’s alpha = 0.895 and Cronbach’s Alpha Based on Standardized Items = 0.898). The corrected item-total correlations were in the acceptable range (0.23 to 0.76 and only two very high correlations of 0.86 and 0.93). Item number 15, assessing the overall experience with the team, did not correlate highly with the rest of the 14 items in the scale (ranges .05 to .44). Cronbach’s Alpha if Item Deleted ranging from 0.87 to 0.89. Thus, we considered reasonable to keep all the items and to report high reliability of the original format for the Romanian scale.

Besides, we have run Chi-square statistics for various demographics to compare the proportion of responses, i.e., “excellent” versus all other answers. Our results show that there are no significant differences in responses among our groups when groups were defined in terms of gender, dwelling, number of previous visits with the physician, type of disease (acute vs. chronic), age, or visiting hospital. We conclude that in this sample, these are not differentiations in terms of responses to communication-focused items.

Furthermore, the most representative aspects raised during the discussions with the patients are described below.

The standard instruction for the patient was ˝Circle your answer for each item below˝. One patient proposed to give an example on how to circle the answers. The team considered that this could influence patients answers. The team added “mark” as it clarifies the action.

For Item 1, two patients would change the word “greeted”. The team decided to switch to “received”, which includes in Romanian the greetings and shows that the patient is welcomed in the medical examination room.

For Item 3, one patient suggested to avoid the repetition of “my (ideas) / my (health)” with “own (health)”. The team decided to keep the initial version as it is less confusing.

For Item 4, three patients added suggestions. The team shortened this item so that it would be easier to understand.

For Item 6, one patient suggested a change to add a timeframe perspective. The team considered as letting somebody to talk doesn’t only mean giving time, but also providing enough attention.

For Item 9, one patient suggested changing the last part with “everything the doctor transmitted to me”. The team decided to keep it as it was, because it is more straightforward, and more comprehensive.

For Item 10, four patients wanted the item to specify the type of questions. The team decided to keep it as it because it is simpler and more comprehensive.

For Item 14, one patient suggested to replace “right” with “adequate”. The team decided to keep the initial version as the suggested word may be more difficult to understand.

Finally, after the harmonization with patient comments and observations the final version of CAT-Ro was obtained (Additional file 2).

## Discussion

The present study represents the first translation and cultural adaptation of CAT in Romanian. Moreover, CAT is the first tool available in Romanian to evaluate the health professionals’ communication skills. We used a rigorous scientific methodology for the translation process, according to literature recommendations, involving conceptual evaluation, semantics, and cultural adaptation. The updated version was pre-tested in a pilot study that included 89 patients to validate it.

To obtain such an instrument, we chose to make a translation and cross-cultural adaptation of a validated tool developed in another language, which is faster and allows us to compare results with international data.

Our results showed a narrow range of variability in item interpretation and a reliable scale for the 14-items of Romanian CAT version. Even though patients’ ability to communicate and understand can be influenced by age, education, type of pathology, duration of relationship with the doctor, in our study no differences were identified in patients’ responses according to such variables.

These results might be explained by the characteristics of the sample finally enrolled in our study, which was not heterogeneous enough from the point of view of the patients’ ability to communicate and understand.

Thus, our group analysis showed a high proportion of educated patients as well as an already existing doctor-patient relationship, which might promote a positive evaluation of the communication. However, the primary objective of our study was to assess the degree of understanding of the translation of this questionnaire, while evaluating the degree of communication with the doctor was only a secondary objective.

This aspect, together with the high degree of concordance registered for each item, represents an additional proof that the CAT is a reliable tool, easy to understand and use.

In the future, we plan to validate CAT_Ro on a more significant number of patients with pathologies from different specialties, both medical and surgical, in other institutions and contexts, to consolidate and extend the use of this tool on a large patient population.

To ensure full cross-cultural correspondence next steps will also need to include a less homogenous sample as per the level of education and previous rate of acquaintance between doctors and patients criteria.

Among the existing tools, CAT was the most appropriate option because it is validated in several languages, easy to use and interpret, easy to understand by patients, the duration is adequate, but at the same time, it is quite complex and comprehensive [26, 28–31].

CAT has also been used in particular circumstances, like how to inform patients about sensitive issues, the occurrence of complications, or discussing an unfavorable prognosis [28]. These circumstances are complex and challenging in clinical practice, in which health professionals must find the balance between the reality that should not be underestimated and the maintenance of hopes. One such example is its use, after translation and cultural adaptation in Portuguese according to the standards recommended in the literature, in a simulated obstetrics and gynecology clinic to assess the announcement of bad news by residents [28]. Even though communicating bad news is a challenge for the doctor-patient relationship, CAT has proven to be a right and valid choice. Moreover, as a result of this study, the department where it took place decided to organize an annual assessment of residents’ interpersonal communication skills.

The 4-Habits Coding Scheme (4-HCS) is a similar initiative of translating into French and adapting cross-culturally another standardized tool to assess physicians’ communication skills. This project used video-recorded consultations as a source for content validation. It has already been translated into different languages [32]. This process also included a good understanding of the 4-HCS concepts by all team members involved in the project, translation quality control, and discussions on translation and harmonization after each stage. The study showed that the French version had internal coherence, which was even higher than the initial US version, which may allow international comparisons. However, the reliability was moderate, requiring the use of two independent evaluators.

Our study was limited to outpatients. For hospitalized patients, the problem of communication with medical staff is different, since they can accept medical decisions much more straightforward. In reality, doctors are the ones who, most of the time, make decisions without involving hospitalized patients [33, 34]. Studies on doctor-patient communication performed on hospitalized patients are less compared to those conducted in the outpatient setting. We can assume that inpatients have serious illnesses, and the decision to initiate investigations and treatment must be made much more promptly by the medical team before the patient can understand his medical situation well enough and express his opinions. An inpatient study showed that although the medical staff explained the care plan to the patients and most believed that the patients had no doubts, the patients interviewed frequently had a different opinion [35]. In this study, hospitalized patients were rarely involved in decision-making and made choices before they expressed their views. There were reported cases in which the patient’s’ decision would not coincide with the doctors’ decision, but there were also patients who did not want to be involved in medical judgments. Thus, these data support the idea that an improvement in communication between hospital health professionals and inpatients can contribute to an increase in the quality of hospital medical services. Because inpatient vs. outpatient doctor-patient communication is different, many medical units use the Hospital Consumer Assessment of Healthcare Providers and Systems (HCAHPS). A systematic review of the literature showed that the HCAHPS score could be improved; more measures are needed to identify problems in and improve communication with inpatients and improve their satisfaction [36].

Improving communication skills is also a requirement for patients [37]. Health professionals would like patients to be able to provide more accurate medical histories, to define symptoms more precisely, to ask more questions about the disease and treatment, to be more honest, ask for clarification, recognize when they have ambiguities, and not to leave important issues for the end of the consultation. Moreover, this study proposes the AGENDA model, which consists of organizing workshops for patients, designed to increase their ability to communicate with medical staff. This model can be used to train patients in this regard, which would increase their confidence in doctors and medical systems, the adherence to treatment, and the quality of healthcare.

There are categories of patients for whom such communication problems are even more complicated; therefore, they must be identified and approached differently. Identifying patients with reduced communication and understanding skills are critical. Consequently, a study aimed to develop and test a new tool, simple and easy to use, designed to help medical staff recognize patients with low abilities in communication and understanding, called “Assess patients’ Communication and Comprehension Skills” (AsCkS) [38]. Challenging communication was found in patients with ages over 65, lower educational levels, or with language difficulties.

Lately, there is a tendency to develop digital tools to achieve better communication, especially with patients suffering from chronic conditions. One such tool is *InvolveMe*, the patient can communicate symptoms, the evolution under treatment, information related to lifestyle, working conditions, health concerns, and emotional and psychological aspects [39]. Using such a tool, before or after the visit, is a complementary way to improve communication between patients and health professionals and paves the way for future research in the direction of digital communication.

After validation, the use of CAT_Ro will have to be extended to other specialties, mainly surgical and primary care, in several situations and circumstances. However, the properties and versatility of the original CAT ensure a great potential and open the way to future research.

## Conclusion

Even in an age when medical, scientific advances are astonishing, medicine remains an art. The communication capacity of health professionals is part of the artistic and human dimensions of medicine. Through interpersonal communication skills, they can obtain better information from patients, facilitate a more accurate diagnosis, and provide optimal treatments. In turn, patients who are pleased with the communication with their doctors will be more confident, motivated, adherent to treatment, and their health outcomes will improve. Studies have shown that there is a frequent discrepancy between doctors’ and patients’ perceptions of communication between them, with doctors tending to overestimate the quality of communication. That is why training in the field of communication has become mandatory in many medical institutions. Improving communication skills implies the availability of tools dedicated to assessing how patients experience the interaction with health professionals.

One such tool is CAT, which has been validated in numerous languages and studies. CAT is a simple tool, easy to use and interpret, it helps manage time effectively, and its results are relevant to how patients appreciate communicating with their physicians.

Our study performed a translation and adaptation of CAT in Romanian, culturally relevant to the Romanian context, following a recommended scientific methodology. The obtained instrument, CAT_Ro, was validated in a pilot study and is ready to be used in more extensive studies with patients from several medical specialties.

## Supplementary Information

**Additional file 1.**

**Additional file 2.**

## Data Availability

All the data and materials used and/or analyzed during the current study are available from the corresponding author.

## Abbreviations

HCP: Healthcare professionals’ communication
CAT: Communication Skills Assessment
CAT_Ro: Romanian version of the CAT
PCVq: Patient-generated questionnaire
4-HCS: 4-Habits Coding Scheme
HCAHPS: Consumer Assessment of Healthcare Providers and Systems
AsCkS: “Assess patients’ Communication and Comprehension Skills”
