# Supplementary material for: Translation and cultural adaptation of a romanian version of the communication assessment tool (CAT_Ro)
Source: BMC Health Serv Res. 2021 Feb 27;21:184. doi: 10.1186/s12913-021-06186-w (PMC7913309; doi:10.1186/s12913-021-06186-w)
Supplement: Supplementary file 1 — Additional file 1. [file 12913_2021_6186_MOESM1_ESM.docx]

| **English** |
| --- |
| **Communication Assessment Tool** |
| Communication with patients is a very important part of quality medical care. We would like to know how you feel about the way your resident physician communicated with you. Your answers are completely confidential, so please be as open and honest as you can. Thank you very much.  **1:** poor **2:** fair **3:** good **4:** very good **5:** excellent  Please use this scale to rate the resident physician’s communication with you.  Circle your answer for each item below. |
| ***The doctor:*** |
| 1. Greeted me in a way that made me feel comfortable |
| 1. Treated me with respect |
| 1. Showed interest in my ideas about my health |
| 1. Understood my main health concerns |
| 1. Paid attention to me (looked at me, listened carefully) |
| 1. Let me talk without interruptions |
| 1. Gave me as much information as I wanted |
| 1. Talked in terms I could understand |
| 1. Checked to be sure I understood everything |
| 1. Encouraged me to ask questions |
| 1. Involved me in decisions as much as I wanted |
| 1. Discussed next steps, including any follow-up plans |
| 1. Showed care and concern |
| 1. Spent the right amount of time with me |
| ***The Doctor’s staff:*** |
| 1. Treated me with respect |
