# Supplementary material for: Translation and cultural adaptation of a romanian version of the communication assessment tool (CAT_Ro)
Source: BMC Health Serv Res. 2021 Feb 27;21:184. doi: 10.1186/s12913-021-06186-w (PMC7913309; doi:10.1186/s12913-021-06186-w)
Supplement: Supplementary file 2 — Additional file 2. [file 12913_2021_6186_MOESM2_ESM.pdf]

NUMELE MEDICULUI:

CHESTIONAR DE EVALUARE A COMUNICĂRII

Comunicarea cu pacienții este o parte foarte importantă a îngrijirii medicale de calitate. Dorim să aflăm ce părere aveți despre modul în care medicul care v-a consultat a comunicat cu dumneavoastră.
Vă asigurăm că toate răspunsurile pe care le veți da sunt complet confidențiale, deci vă rugăm să fiți cât se poate de deschis(ă) și sincer(ă). Vă mulțumim foarte mult.

Vă rugăm să utilizați opțiunile de mai jos pentru a evalua comunicarea medicului cu dumneavoastră. Încercuiți un singur răspuns pentru fiecare propoziție de mai jos.

**Medicul:**	Slabă	Destul de bună	Bună	Foarte bună	Excelentă
1. M-a primit într-un mod care m-a făcut să mă simt confortabil	1	2	3	4	5
2. A fost respectuos cu mine	1	2	3	4	5
3. A fost interesat de părerile mele despre sănătatea mea	1	2	3	4	5
4. A înțeles principalele mele motive de îngrijorare legate de sănătatea mea	1	2	3	4	5
5. Mi-a acordat atenție (m-a privit, m-a ascultat atent)	1	2	3	4	5
6. M-a lăsat să vorbesc fără să mă întrerupă	1	2	3	4	5
7. Mi-a dat toate informațiile pe care le doream	1	2	3	4	5
8. A folosit cuvinte pe care le-am înțeles	1	2	3	4	5
9. S-a asigurat că am înțeles tot ceea ce trebuia	1	2	3	4	5
10. M-a încurajat să pun întrebări	1	2	3	4	5
11. M-a implicat în luarea hotărârilor, așa cum mi-am dorit	1	2	3	4	5
12. A discutat cu mine toți pașii următori, inclusiv planurile viitoare de îngrijire	1	2	3	4	5
13. Mi-a arătat grijă și implicare	1	2	3	4	5
14. A petrecut cu mine o perioadă de timp potrivită	1	2	3	4	5
**Ceilalți membri din personalul medical**					
15. Au fost respectuoși cu mine	1	2	3	4	5

COMENTARII:

****

~ Se continuă pe pagina următoare ~

CAT_Ro - Copyright © 2020 – Gregory Makoul, PhD – Toate drepturile rezervate – permis pentru uz non-comercial, educațional

Întrebările de mai jos au un scop statistic. Răspunsurile dumneavoastră vor rămâne anonime.

1. Vârsta dumneavoastră în ani împliniți:
□1 sub 24
□2 25-44
□3 45-64
□4 64-84
□5 peste 85

2. Sunteți :
□1 Femeie
□2 Bărbat

3. Ați mai fost consultat de acest medic înainte?
□1 Nu
□2 Da, dar o singură dată
□3 Da, de mai multe ori

4. Care este limba dumneavoastră maternă?
□1 Română
□2 Maghiară
□3 Altă limbă. Vă rugăm să precizați: ____________________________

5. Ultima școală absolvită/nivel de educație:
□1 Nu am fost la școală
□2 Școală primară (4 clase)
□3 Gimnaziu (8 clase)
□4 Liceu
□5 Școală profesională
□6 Școală postliceală
□7 Studii universitare
□8 Studii post-universitare

6. Mediul dvs de proveniență este:
□1 Rural
□2 Urban

7. Boala de care suferiţi este:
□1 Acută
□2 Cronică

8. Dumneavoastră ați fost azi pacientul:
□1 Da
□2 Nu, eu am acompaniat pacientul

[2]

CAT_Ro - Copyright © 2020 – Gregory Makoul, PhD – Toate drepturile rezervate – permis pentru uz non-comercial, educațional
